# A Case of Repeated TAFRO Syndrome-Like Symptoms and Retroperitoneal Hemorrhage in a Patient With Sjögren Syndrome

**DOI:** 10.7759/cureus.12175

**Published:** 2020-12-19

**Authors:** Takanori Ohta, Naoki Oda, Keiko Saito, Sadafumi Tamiya, Toshiyuki Ueno

**Affiliations:** 1 Department of Internal Medicine, Kitakyushu Municipal Medical Center, Kitakyushu, JPN; 2 Department of Pathology, Kitakyushu Municipal Medical Center, Kitakyushu, JPN

**Keywords:** multicentric castleman disease, tafro syndrome, sjögren syndrome, microangiopathy, tocilizumab, rituximab, tma, retroperitoneal hemorrhage, autoimmune diseases

## Abstract

A 50-year-old Japanese man complaining of dry mouth and eyes, pale skin with cold irritation, and worsening epigastric pain was admitted to the hospital, whereupon he developed fever and anasarca. A computed tomography (CT) scan showed ascites, hepatosplenomegaly, and mildly enlarged multiple lymph nodes, and blood examination revealed renal impairment, thrombocytopenia, and high levels of C-reactive protein (CRP). He was diagnosed with Sjögren syndrome and concurrently manifested symptoms resembling TAFRO syndrome (i.e., thrombocytopenia (T), anasarca (A), fever (F), reticulin fibrosis (R), and organomegaly (O)). Although the TAFRO syndrome-like symptoms progressed, he gradually recovered with immunosuppressive agents. Seven years and five months after the admission, the TAFRO syndrome-like symptoms recurred. Bone marrow biopsy specimens revealed reticulin fibrosis. Inguinal and mediastinal lymph nodes biopsy specimens revealed Castleman disease-like features. Although the symptoms indicated TAFRO syndrome, a diagnosis was not possible owing to the presence of hypergammaglobulinemia and Sjögren syndrome, which required exclusion. Corticosteroid treatment was initiated; however, it was complicated by retroperitoneal hemorrhage, probably due to microangiopathy. After additional treatment with tocilizumab and rituximab, the TAFRO syndrome-like symptoms improved and the hemorrhage progression stopped. In conclusion, TAFRO syndrome-like symptoms may recur with vascular complications and can be successfully treated with tocilizumab and rituximab during Sjögren syndrome. The etiology of TAFRO syndrome could potentially involve Sjögren syndrome, and these syndromes may co-exist.

## Introduction

TAFRO syndrome is a recently recognized systemic inflammatory disorder, characterized by thrombocytopenia (T), anasarca (A), fever (F), reticulin fibrosis (R), and organomegaly (O). Its diagnostic criteria, disease severity classification, and treatment strategy were proposed by Masaki et al. in 2015 [1]. According to the updated 2019 diagnostic criteria for TAFRO syndrome, Sjögren syndrome has been newly added to the exclusion criteria during differential diagnosis [2]. However, patients with a definitive diagnosis of Sjögren syndrome or those positive for Sjögren's antibodies but with an uncertain diagnosis have reportedly manifested the clinicopathological features of TAFRO syndrome [3-7]. Cases of human herpes virus-8-negative multicentric Castleman disease (MCD) of unknown etiology are referred to as idiopathic MCD (iMCD). Positive cases of anti-Sjögren's syndrome-related antigen A (anti-SS-A) antibody have been reported in iMCD, of which TAFRO-iMCD syndrome is considered a subtype [8-10]. Thus, TAFRO syndrome could be associated with Sjögren syndrome. However, the relationship between these two syndromes is still unclear and its differential diagnosis remains challenging. Therefore, this case, which presented repeated TAFRO syndrome-like symptoms and hemorrhage related to thrombotic microangiopathy (TMA) during Sjögren syndrome, provides valuable information about the association between and etiology of the two syndromes.

## Case presentation

A 50-year-old Japanese man with a medical history of sinusitis and hyperuricemia complained of dry mouth and eyes from September 2011, and pale skin on cold irritation and epigastric pain from December 2011. Because over-the-counter medications were not effective for the progressing epigastric pain, he visited a neighboring general hospital in January 2012 where a computed tomography (CT) scan showed mildly enlarged mediastinal and aortic lymphadenopathy, and blood examination showed elevated creatinine (Cre) level (1.39 mg/dL), thrombocytopenia (9×10^4^/μL), and a high level of C-reactive protein (CRP) (13.05 mg/dL). Considering that no apparent gastrointestinal, cardiac, or infectious disease could be identified, as well as the presence of dryness symptoms and Raynaud's phenomenon, the patient was referred to a department of rheumatology in a larger general hospital. After admission to the larger hospital in February 2012, he developed a high fever. His physical examination revealed abdominal tenderness, anasarca, dry mouth and eyes, and symptoms typical of Raynaud's phenomenon. The results of the laboratory examinations are presented in Table 1.

**Table 1:** Laboratory findings at onset and relapse Abbreviations: Anti-SS-A/B: Anti-Sjögren's-syndrome-related antigen A/B, NA: not analyzed, MPO-ANCA: Myeloperoxidase-anti neutrophil cytoplasmic antibody, PR3-ANCA: Proteinase 3 anti-neutrophil cytoplasmic antibody, SIL-2R: Soluble interleukin-2 receptor, IL-6: Interleukin-6, VEGF: Vascular endothelial growth factor, HHV-8: Human herpesvirus-8, HIV: Human immunodeficiency virus, CMV: Cytomegalovirus, EBV: Epstein-Barr virus

Situation			onset	relapse
	Admission year		2012	2019
Blood examinations (reference range)				
	White blood cells, μL		8700	3000
	Neutrophils, %		81	27
	Lymphocytes, %		10	53
	Monocytes, %		9	13
	Eosinophils, %		0	5
	Basophils, %		0	2
	Red blood cells, 10^6^/μL		502	305
	Reticulocytes, %		6	4
	Hemoglobin, g/dL		15.8	9.9
	Platelets, 10^4^/μL		8.1	4.4
	Minimum platelet count		0.3	1.9
	Aspartate aminotransferase, IU/L		19	12
	Alanine aminotransferase, IU/L		18	4
	Lactate dehydrogenase, IU/L		236	145
	Alkaline phosphatase, IU/L		1084	436
	γ-glutamyl transpeptidase, IU/L		188	31
	Total protein, mg/dL		6.5	7.6
	Albumin, mg/dL		3	3.4
	Total-bilirubin, mg/dL		0.6	1.1
	Creatinine, mg/dL		1.04	1.11
	Maximum creatinine value		5.83	3.79
	Blood urea nitrogen, mg/dL		27	19.4
	Creatine kinase, IU/L		21	31
	Ferritin, ng/mL		218	297.9
	C-reactive protein, mg/dL		25.72	1.282
	Maximum c-reactive protein value		29.47	2.318
	IgG, mg/dL		1024	2500
	IgA, mg/dL		214	329
	IgM mg/dL		244	337
	Anti-nuclear antibody		<40	<40
	Anti-SS-A* antibody, U/mL		negative	negative
	Anti-SS-B* antibody, U/mL		negative	negative
	Anti-mitochondrial M2 antibody (<7)		181	551
	Rheumatoid factor, IU/mL		negative	negative
	Anti-DNA antibody, IU/mL		NA*	negative
	Anti-double-stranded DNA antibody		negative	negative
	Anti-Smith antibody		negative	negative
	Platelet associated IgG, ng/10^7^ cells		237	237
	Direct Coombs test		negative	negative
	Indirect Coombs test		negative	negative
	Lupus anticoagulant		negative	negative
	Anti-cardiolipin antibody		negative	negative
	MPO-ANCA*		negative	negative
	PR3-ANCA*		negative	negative
	SIL-2R*, U/mL (<534)		1148	1360
	IL-6*, pg/mL (<4)		NA	15.1
	VEGF*, pg/mL		NA	70
	HHV-8* DNA		NA	negative
	HIV* antibody		negative	negative
	CMV*-IgM (<0.8)		NA	2.01
	CMV-IgG (<2)		NA	23.6
	CMV antigenemia		negative	negative
	EBV* capsid antigen-IgG (<10)		20	NA
	EBV capsid antigen-IgM (<10)		<10	<10
	EBV nuclear antigen (<10)		40	NA
Urine examinations				
	Protein		1+	2+
	Occult blood		negative	2+
	β2-microglobulin, ng/mL		2257	5698.3

**Figure 1:**
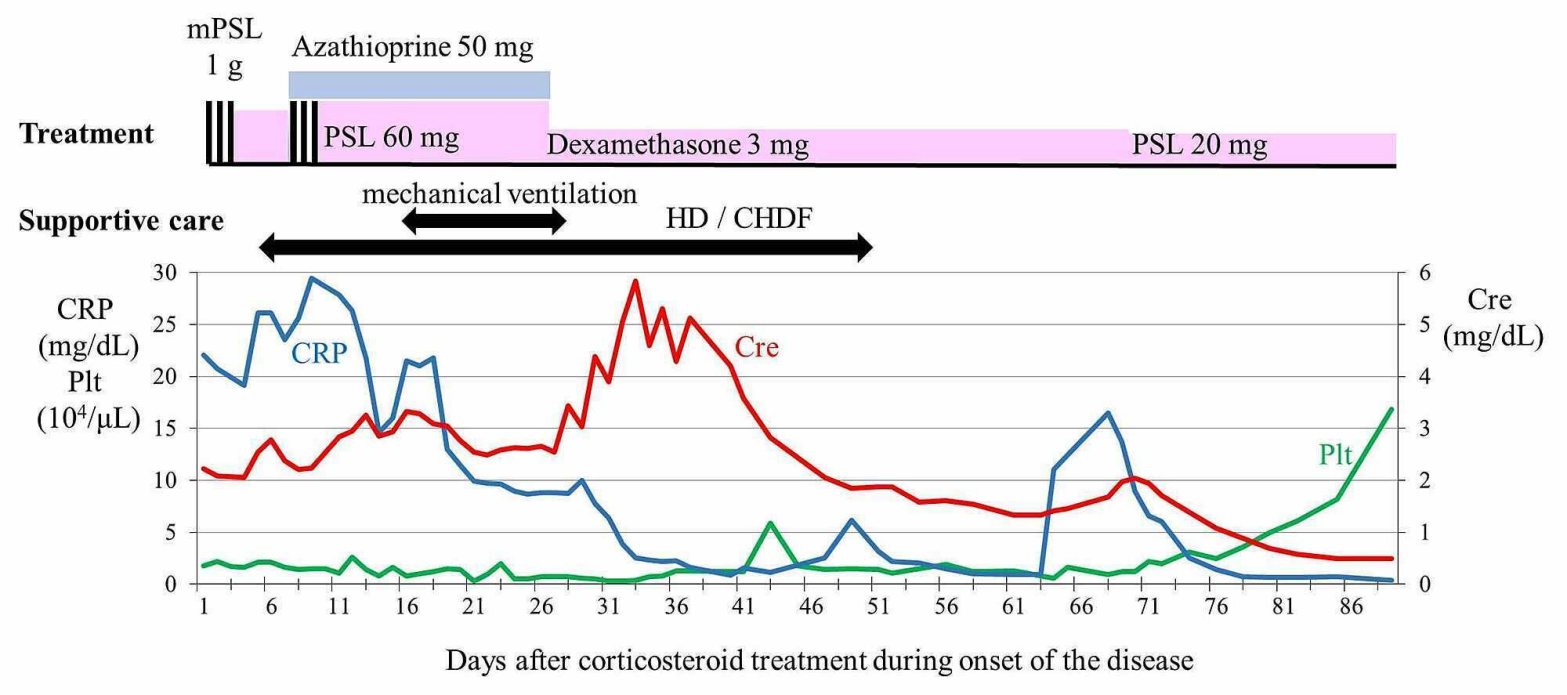
Clinical course of onset Abbreviations: mPSL: Methylprednisolone, PSL: Prednisolone, HD: Hemodialysis, CHDF: Continuous hemodiafiltration, CRP: C-reactive protein, Cre: Creatinine, Plt: Platelet

A CT scan showed ascites, hepatosplenomegaly, and mildly enlarged lymph nodes in the inguinal, mediastinal, and para-aortic regions. Inguinal lymph node biopsy specimens did not provide a definitive diagnosis such as malignant lymphoma. No infectious diseases responsible for the elevated CRP could be clinically detected. Lip biopsy of his labial salivary gland showed focal lymphocytic sialadenitis and a focus score ≥of 1 foci/4 mm^2^, and the unstimulated whole saliva flow rate was 0.08 mL/minute. Based on those findings, a diagnosis of Sjögren syndrome was made, although antibodies for anti-SS-A and anti-Sjögren's-syndrome-related antigen B (anti-SS-B) were negative. In addition, the patient had symptoms of dry mouth and eyes lasting six months, and no disease other than Sjögren syndrome was identified as causing the sicca syndrome. Increased levels of alkaline phosphatase (ALP) and positivity for the anti-mitochondrial M2 antibody were related to subclinical primary biliary cholangitis. These symptoms could be explained as an acute exacerbation of Sjögren syndrome with subclinical primary cholangitis. However, the patient’s symptoms exhibited aggressive clinical behavior compared to the typical course of Sjögren syndrome. Therefore, complications of other autoimmune diseases, such as vasculitis, were suspected but not identified. Corticosteroid pulse followed by high-dose corticosteroid was administered (starting from day 1) as first-line therapy, but it had no dramatic impact on the patient’s epigastric pain, anasarca, fever, or abnormal blood results, including the CRP level, Cre level, and thrombocytopenia. Furthermore, pleural effusion, which had serous characteristics without pathogens and atypical cells, appeared and increased. Dialysis for progressive anasarca and renal dysfunction was initiated on day 6, as shown in Figure 1.

Although azathioprine was added, the patient developed respiratory failure due to large amounts of pleural effusion that required mechanical ventilation and intrathoracic drainage on day 16. Appropriate antibiotics and antivirals were concurrently administered in anticipation of possible secondary infections. Blood, urine, and pleural fluid cultures were examined as deemed necessary; if infection was considered, antibiotics were administered, or catheter replacements were performed according to infection susceptibility. However, the patient progressed to multi-organ failure. While continuing corticosteroid treatment with these multidisciplinary support modalities, artificial respiration and renal replacement therapy were withdrawn on day 28 and day 52, respectively. Beginning on days 71 and 78 of the corticosteroid treatment, there was a trend of improvement without dialysis in serum Cre level and platelet count, respectively. High inflammatory response with elevated CRP level finally improved, the anasarca disappeared, and the patient was discharged after a total of 104 days of hospitalization. Subsequently, the patient was followed up by a rheumatologist near his home, and the corticosteroid dosage was tapered off until January 2013, for a total of 12 months of corticosteroid treatment. The patient chose not to continue further follow-up hospital visits because his symptoms completely resolved, except for mild Raynaud's phenomenon and sicca syndrome.

**Figure 2:**
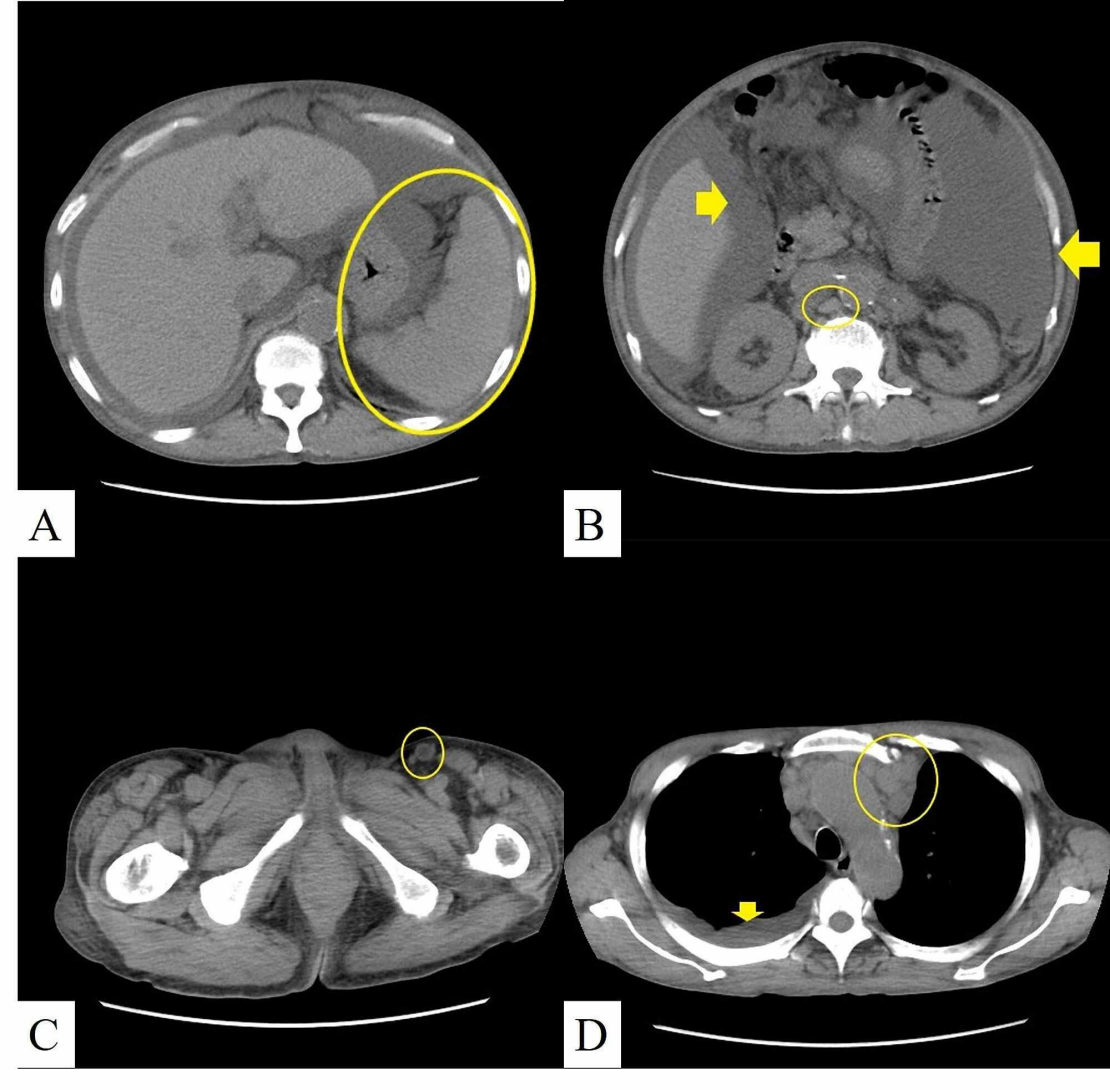
A computed tomography scan revealed splenomegaly (circle) (A); massive ascites (arrows); mildly enlarged lymph nodes in the para-aorta (circle) (B); inguinal (circle) (C), and mediastinal regions (circle); and pleural effusion (arrow) (D)

In July 2019, seven years and five months after the initial admission, the epigastric pain recurred. In November 2019, he was admitted to our hospital because of worsening epigastric pain and pyrexia. His physical examination revealed abdominal distension, bilateral pitting pedal edema, and a mildly enlarged inguinal lymph node. The laboratory examination results are presented in Table 1. A CT scan revealed splenomegaly, massive ascites, mildly enlarged para-aortal, inguinal, and mediastinal lymph nodes; and pleural effusion (Figures 2A-2D).

**Figure 3:**
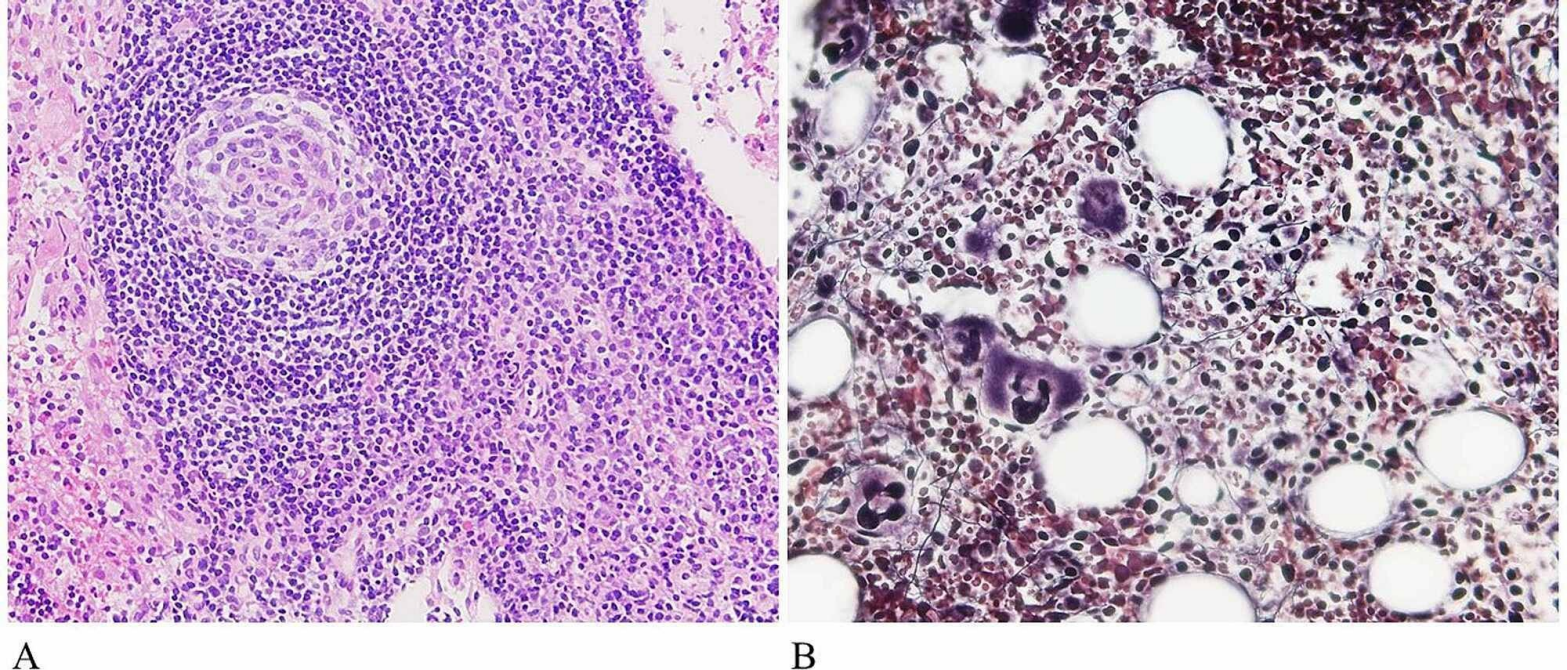
Biopsy specimen from the left inguinal lymph node showing an atrophic germinal center surrounded by increased scattered plasma cells (Hematoxylin & Eosin, ×400) (A). Bone marrow biopsy specimen showing an increased number of megakaryocytes and reticulin fibrosis (Gitter staining, ×400) (B)

Biopsy specimens from the left inguinal and mediastinal lymph nodes revealed atrophic germinal centers surrounded by increased scattered plasma cells, which were indicative of Castleman disease but made the definitive diagnosis of malignant lymphoma or typical TAFRO syndrome difficult (Figure 3, Panel A).

Bone marrow biopsy specimens revealed an increase in the number of bone marrow megakaryocytes and reticulin fibers, as shown in Figure 3 (Panel B). His ascites had serous characteristics. No pathogen was detected by culture, and no atypical cells were observed on cytological examination. The clinical symptoms and laboratory findings in November 2019 were like those in February 2012 and mimicked the features of TAFRO syndrome (Tables 1-2 and Figures 2-3).

**Table 2:** Summary of symptoms during the course of Sjögren syndrome

Years (situation)	2012 (onset)	2019 (relapse)
Findings resembling TAFRO syndrome	・Thrombocytopenia ・anasarca ・systemic inflammation ・mild organomegaly ・progressive renal insufficiency ・elevated level of serum alkaline phosphatase	・Thrombocytopenia ・anasarca ・systemic inflammation ・mild organomegaly ・progressive renal insufficiency ・elevated level of serum alkaline phosphatase ・beneficial effects of tocilizumab and rituximab
Findings unlike TAFRO syndrome	・Raynaud's phenomenon ・sicca syndrome with focal lymphocytic sialadenitis from labial salivary gland and low unstimulated whole saliva flow rate ・positive for anti-mitochondrial M2 antibody	・Raynaud's phenomenon ・sicca syndrome ・positive for anti-mitochondrial M2 antibody ・elevation to hypergammaglobulinemia

**Figure 4:**
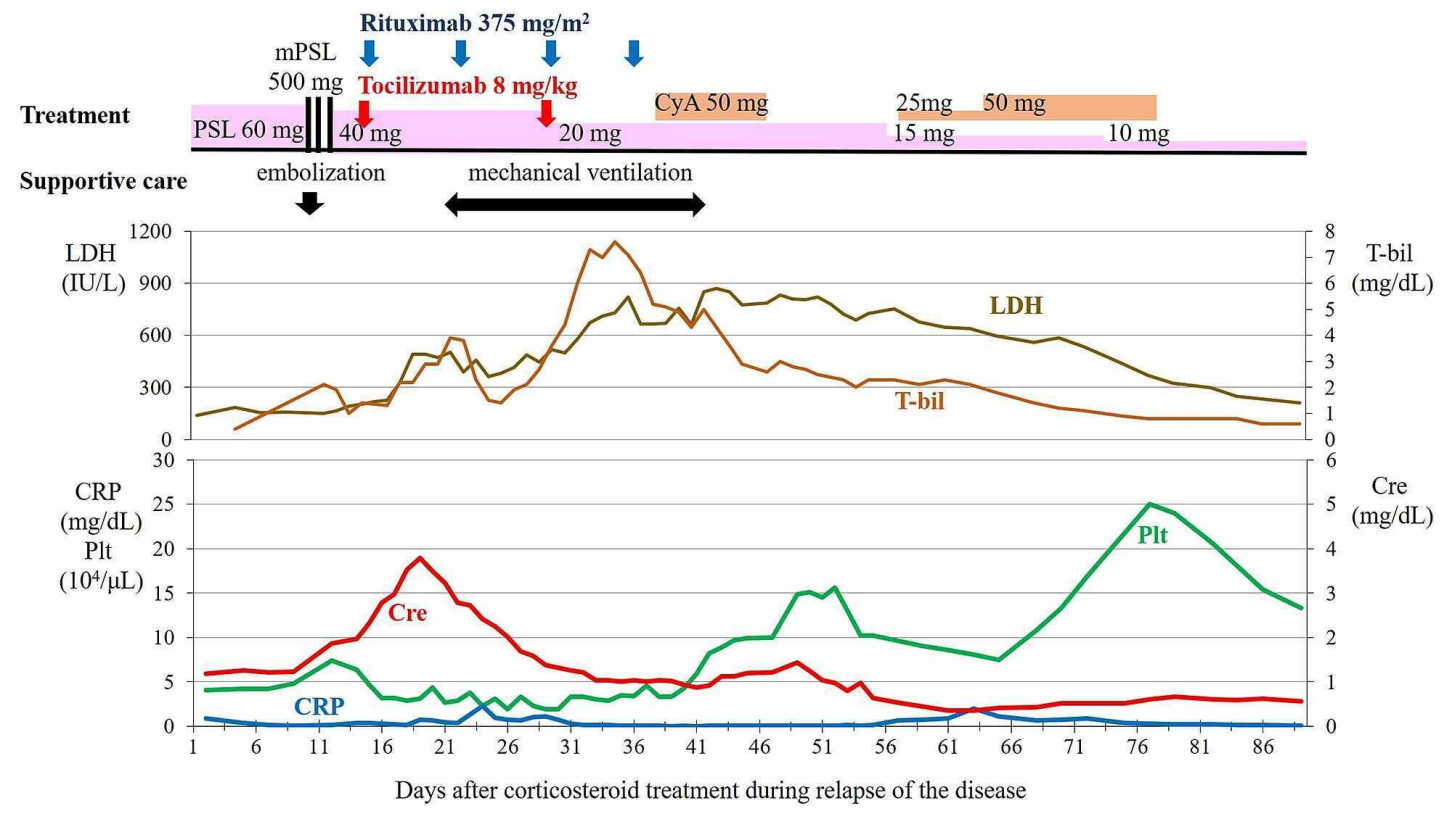
Clinical course of relapse Abbreviations: mPSL: Methylprednisolone, CyA: Cyclosporine, PSL: Prednisolone, LDH: Lactate dehydrogenase, T-bil: Total bilirubin, Cre: Creatinine, Plt: Platelet, CRP: C-reactive protein

Therefore, we re-evaluated the patient’s symptoms from 2012 because TAFRO syndrome was an unrecognized disorder at that time. However, our findings confirmed a definitive diagnosis of primary Sjögren syndrome based on the classification criteria of the American College of Rheumatology (ACR) and the European League Against Rheumatism (EULAR). In addition, the laboratory abnormalities observed in 2019, including elevated gammaglobulinemia (IgG 2500 (normal range; 861～1747) mg/dL) and positive mitochondrial M2 antibodies are generally found in Sjögren syndrome and not in TAFRO syndrome [11]. Considering that a diagnosis of TAFRO syndrome requires the exclusion of Sjögren syndrome and the absence of hypergammaglobulinemia [2,8], we concluded that his disease was a Sjögren syndrome with TAFRO syndrome-like symptoms. Although cytomegalovirus IgM was positive on admission, the intravenous administration of ganciclovir was ineffective. Thereafter, a high-dose corticosteroid treatment regimen was initiated on day 1. Pyrexia improved rapidly, but renal dysfunction, ascites, and thrombocytopenia did not respond, as shown in Figure 4.

**Figure 5:**
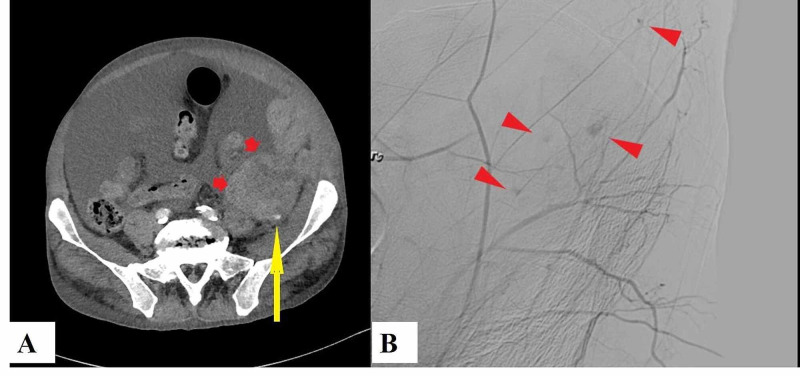
Contrast computed tomography scan showing extravasation (long arrow) around the retroperitoneal hematoma (short arrows) (A). Angiographic examination revealed multiple extravasations (arrowheads) in the peripheral vessels (B)

On day 10, he developed mild disorientation and severe abdominal distention, and the characteristic of ascites changed from serous to bloody. The hemoglobin level decreased rapidly, and a contrast CT scan revealed the presence of extravasations with a retroperitoneal hematoma in a large amount of ascites, as shown in Figure 5 (Panel A).

It was clear that the hemorrhage was not caused by the ascites puncture procedures because of the different locations. An emergency angiography revealed multiple microangiopathies of the left peripheral vessels of the iliac lumbar, lumbar, and iliac arteries, which likely caused peritoneal bleeding (Figure 5, Panel B). The angiography revealed no aneurysms or arterial dissections. Although embolization of these bleeding regions was performed and a methylprednisolone pulse (500 mg/day for three days) was started, the hematoma spread into the ascites and the TAFRO symptoms did not improve. At the same time, the platelet count and coagulation examination results were as follows: decreased levels of platelet count (7.4×10^4^ /µL) and fibrinogen (137 mg/dL); a slightly elevated level of fibrinogen degradation products (11.1 μg/mL); slightly prolonged prothrombin time (international normalized ratio: 1.26); and a disintegrin and metalloproteinase with a thrombospondin type 1 motif, member 13 (ADAMTS 13), was within the normal range of activity (53%). Peripheral blood smears did not show remarkable fragmentation of the red blood cells. The patient was in a critical condition with progressive renal dysfunction, making adequate use of immunosuppressive drugs, such as cyclosporine and azathioprine, difficult. After written informed consent was obtained from the patient and his wife, additional treatment with intravenous tocilizumab (8 mg/kg, once every two weeks beginning from day 14) and rituximab (375 mg/m^2^, once a week from day 15), without the need to adjust the dose due to renal dysfunction, was initiated in addition to prednisolone based on the proposed treatment for TAFRO syndrome [1]. His consciousness progressively worsened from day 17. In parallel, there were gradual increases in serum total bilirubin (T-bil) (indirect bilirubin dominant) and lactate dehydrogenase (LDH) levels and decreases in haptoglobin level (<10 mg/dL), which indicated a new complication of hemolytic anemia as shown in Figure 4. The patient’s renal dysfunction, thrombocytopenia, and microangiopathy were accompanied by a disturbance in the level of consciousness, hemolytic anemia, and LDH elevation, suggesting the development of TMA. Thereafter, he developed severe aspiration pneumonia against a background of confusion and required a mechanical ventilator on day 21. With repeated administration of tocilizumab and rituximab plus the continuation of corticosteroid and supportive treatment, serum Cre showed a trend of improvement from day 19, T-bil from day 35, and platelets from day 39. Similarly, his breathing improved, and he was able to discontinue ventilatory support on day 41. Anemia also improved and red blood cell transfusions were no longer needed from day 31. Although the amount of ascites was markedly reduced, the hematoma with a small amount of ascites remained. Similarly, the lymph node enlargement was reduced but did not disappear. The tapered prednisolone normalized laboratory serum data, such as the platelet count, Cre, CRP, T-bil, and LDH. The patient was doing well and was rehabilitated and discharged home after a total of 102 days in our hospital. During the 10 months outpatient follow-up period, the patient experienced no symptom recurrence with 5 mg of oral prednisolone.

## Discussion

The symptoms in this present case closely resembled TAFRO syndrome in terms of clinicopathological findings, aggressive clinical course, and beneficial effects of tocilizumab and rituximab [1-2,6-7,12]. However, according to the diagnostic criteria for TAFRO syndrome proposed by Masaki et al., TAFRO syndrome can only be diagnosed after excluding Sjögren syndrome, and the thrombocytopenia, anasarca, fever, myelofibrosis, organomegaly, and renal insufficiency in this patient can be accounted for by Sjögren syndrome [2,11,13-14]. Similarly, according to the diagnostic criteria proposed by Iwaki et al., the absence of hypergammaglobulinemia is a major criterion for the diagnosis of TAFRO-iMCD. Therefore, we concluded that this patient was diagnosed as having Sjögren syndrome with TAFRO syndrome-like symptoms. There are previous case reports and studies of patients with a definitive diagnosis of Sjögren syndrome or those positive for anti-SS-A and/or anti-SS-B antibodies but with an uncertain diagnosis that developed clinical features of TAFRO syndrome, and some of them relapsed [3-7]. In this case, TAFRO syndrome-like symptoms, such as thrombocytopenia, anasarca, fever, myelofibrosis, organomegaly, and renal insufficiency also occurred with Sjögren syndrome. These results suggest that the etiology of TAFRO syndrome may be associated with Sjögren syndrome. Furthermore, TAFRO syndrome may co-exist with Sjögren syndrome. More recently, increased serum interferon γ-induced protein 10 (IP-10), a cytokine belonging to the CXC chemokine family, was observed in patients with TAFRO syndrome during flare-ups [15]. Considering that the expression of IP-10 has been associated with autoimmune diseases, including Sjögren syndrome; inflammatory diseases, including viral, bacterial, and mycotic infections; immune dysfunction; and tumorigenesis; TAFRO syndrome develops in association with these diseases. Interestingly, in this case, the levels of CRP at relapse were not increased as compared to the onset during Sjögren syndrome, as shown in Table 1, Figure 1, and Figure 4. Hence, we hypothesize that the TAFRO symptoms in this patient may be caused by different IP-10-expressing factors. For example, we can assume that the trigger at onset might be a bacterial infection and a trigger at relapse might be a viral infection, resulting in different CRP levels with a background of Sjögren syndrome. The etiology of TAFRO syndrome and the association between Sjögren syndrome and TAFRO syndrome are still unclear. It is expected that biomarkers specific to TAFRO syndrome will be identified in the future.

In this case, the retroperitoneal hemorrhage may have been related to TMA, although other causes, such as vascular weakness, thrombocytopenia, and disseminated intravascular coagulation, might have also been related. The most common autoimmune disorders complicating secondary TMA are systemic lupus erythematosus and systemic sclerosis; there are also reports of Sjögren syndrome complicating TMA [16-17]. In TAFRO syndrome, renal biopsies often show TMA-like findings that may be crucial to the development of acute renal dysfunction [5]. Additionally, tocilizumab was also reported to cause an adverse event of multifocal microangiopathy [18]. The bleeding and disorders of consciousness, in this case, occurred prior to the first infusion of tocilizumab, suggesting that tocilizumab was not the primary cause of TMA. Secondary TMA has been reported to be caused by a variety of triggers, including autoimmune diseases, infections, drugs, malignancies, transplants, and pregnancy [19]. Therefore, it is difficult to identify a specific factor, and this case may have involved multiple factors, including Sjögren syndrome, TAFRO symptoms, tocilizumab, as well as others.

The patient's relapse symptoms recovered earlier and were less severe than those at onset (Figure 1 and Figure 4). We considered this amelioration of symptoms to be the result of the early diagnosis of relapse and rapid therapeutic intervention, the previously mentioned different triggers hypotheses, and the effectiveness of additional therapies with tocilizumab and rituximab. Recently, tocilizumab or rituximab for primary Sjögren syndrome has failed to show significant advantages in randomized double-blind controlled trials, although these assessed outcomes were different from TAFRO symptoms [20]. On the contrary, tocilizumab, rituximab, or both have been reported as effective and well-tolerated in case reports of TAFRO syndrome and have been recommended as a treatment option [1,4-7,12]. A standard regimen for the order, methods, and duration of the administration of tocilizumab and rituximab has not been established. However, the coadministration of tocilizumab, rituximab, and corticosteroid has been used in first-line treatment, in relapse, and in critical situations, and successful responses to the treatment have been reported [5,12]. This presented case was in a recurrent, corticosteroid-resistant, and life-threatening situation. Furthermore, because renal replacement therapy using anticoagulants was not feasible due to the risk of worsening retroperitoneal hemorrhage, we decided to use coadministration therapy, expecting an immediate therapeutic effect. As a result, the coadministration therapy at relapse resulted in early response and recovery of TAFRO symptoms as compared to the symptoms at the onset. In addition, after these therapies, the progression of retroperitoneal hemorrhage stopped. Blood examinations, including T-bil and LDH, eventually normalized. Coadministration therapy using tocilizumab, rituximab, and corticosteroid may have been effective for hemorrhage probably due to TMA as well as TAFRO syndrome-like symptoms during Sjögren syndrome.

## Conclusions

The TAFRO syndrome-like symptoms during Sjögren syndrome closely resembled TAFRO syndrome in terms of the clinicopathological findings, aggressive clinical course, and beneficial effects of tocilizumab and rituximab. The etiology of TAFRO syndrome could potentially involve Sjögren syndrome, and these syndromes may be co-existing. A careful follow-up of Sjögren syndrome would be necessary because life-threatening TAFRO symptoms may recur and lead to vascular complications related to TMA. Accumulation of similar cases and further research are needed.
